# It’s a pain in the neck! Who can help me?

**DOI:** 10.1186/2045-4015-2-43

**Published:** 2013-11-18

**Authors:** Gillian Schiller

**Affiliations:** 1 Partner, Gateway Consulting Group, 14 Cecil Park, Pinner Middx HA5 5HH, UK

## Abstract

Orthopaedic surgery is a long established surgical specialty. Practitioners complete training which focuses on those musculo-skeletal conditions for which surgery is the preferred treatment option. Diagnosis and assessment are performed to exclude those for whom surgical intervention is not indicated. Finestone and others argue in their recent IJHPR paper that we need a subspecialty to cater for these patients – medical orthopaedics. They explain that almost all of the very many patients who seek help for musculo-skeletal conditions do not need surgery, and that orthopaedic surgeons are ill equipped to deal with their complaints and have little to offer. Their paper sets out the case for a new discipline of medical orthopaedics to complement the discipline of orthopaedic surgery.

This commentary examines the case for a new approach to musculo skelketal conditions. It discusses the steps which might be needed to decide whether any new discipline should be established, and goes on to set the terms for a debate on the merits of a multidisciplinary approach compared to a purely medical one. Leadership in clinical disciplines has historically rested with medically qualified health professionals. Nowadays this has given way to an understanding that health problems, especially long term conditions, commonly benefit from different expertises coming together. For musculoskeletal conditions, might this be an example where the best outcome would be to look beyond a medically led model?

This is a commentary on http://www.ijhpr.org/content/2/1/42/.

## Commentary

### Why is this issue important?

Worldwide the burden of illness from musculo skeletal conditions (MSC) is very large. Spending on investigations and treatments is concomitantly sizeable. Many standard aides and devices are expensive and lack an evidence base for their efficacy. In an article in this journal written by Finestone and colleagues they call for a better approach to the assessment and treatment of the high proportion of MSC sufferers where patients would not benefit from surgical treatment [1]. They propose establishing a new medical specialty – medical orthopaedics - that would better tackle the needs of this group of patients.

Before examining the case to be made for a new approach to non-surgical treatment of MSC, it is worth estimating the scale of the problem. Consider the level of expenditure by three nations (all sums are in US$), using expenditure on cardiovascular disease as a reference point. In 2008, Australia estimated that it spent about $ 5.7 Billion (US $) on musculoskeletal disorders, and approaching $ 8 Billion (US$) on cardiovascular disease [2]. In 2012, the UK NHS spent over $8 Billion (US$) on musculo skeletal conditions, and $ 11 Billion (US$) on circulatory disorders [3]. In the USA estimates showed that patients with musculoskeletal conditions incurred total annual medical care costs of approximately $240 billion, of which $77 billion was directly related to the musculoskeletal conditions and the remainder to indirect costs [4]. According to a 2006 review, total costs associated with low back pain in the United States exceeded $100 billion that year, two thirds of which were a result of lost wages and reduced productivity [5]. Coronary heart disease costs the USA approximately $110 billion each year [6]. These estimates of spend demonstrate that the resources committed to MSC are comparable with the funds devoted to circulatory illness and are, using typically English understatement, substantial.

The question of why there is no delineated medical specialty focusing on orthopaedic medicine isn’t original – indeed I recall debating it when a medical student myself. The response to the question during my student days was that the area was covered by physiotherapists, to whom physicians and surgeons alike could refer. The paper by Finestone and colleagues argues that the MSC complaints patients bring are commonplace and at present poorly handled by clinical professionals. They propose training medically qualified specialists to properly diagnose and set out paths of treatment, and to gather a body of evidence for the most effective actions for any given set of symptoms and signs.

### What do we know?

We know that MSC needing non-surgical interventions are common. We know that there is no established, dedicated medical specialty to which patients can turn. The authors believe that the void presented by orthopaedics being an essentially surgical-only specialty impedes proper diagnosis and appropriate treatment for the great majority of people with MSC.

Patients seek help from a range of people they believe have useful expertise, usually including at least some of family doctors, rheumatologists, neurologists, rehabilitation specialists, gait specialists, physiotherapists, and orthopaedic surgeons. They take a seemingly random route on their journey to find symptom relief, shuttling between one clinical specialty and another, sometimes seeing several members of one clinical discipline before discarding it in favour of another. The available sources of help are not organised so as to guide people in deciding which might be of greatest benefit. How do they know which conditions deserve a visit to a rheumatologists, but only after the family doctor has ruled out conditions not dealt with by that specialty? People often spend a good deal of either their own money or that of their insurers to little effect. They seek relief from pain and improvement of function but rarely achieve these goals easily. Dissatisfied, they try various clinicians, sometimes including people trained in less well accredited disciplines such as hypnosis, and sometimes people many might label as medical charlatans.

All this activity is only enjoyed by those who have insurance or the means to pay for their appointments and treatments. There is a real ethical case to be made for exploring this area of unmet need based on the strong likelihood that people from the poorest backgrounds, who commonly have worse health than those with greater wealth, do not even have the opportunity to remedy their suffering at present.

The authors highlight the extensive use of inappropriate investigations carried out partly because insurers will pay for them, and partly under pressure from patients themselves. Patients commonly believe that imaging or blood tests will lead to a correct diagnosis and will, at the very least, allow the clinician to rule out sinister conditions like cancer – a very real fear for many people and a key reason offered when they are asked why they seek a specialist opinion.

Imaging is only helpful when used against strict criteria, but the fact that it might be covered by insurers means it is used far more widely than appropriate. Protocols containing evidenced criteria have been developed in many places, including examples from the UK [7,8]. Adhering to them cuts the numbers of imaging requests made and fulfilled, saving patients anxiety and the health system a great deal of money, without any loss of quality or difference to outcomes. All imaging techniques have some risk. Concomitant benefit must be shown where imaging is to be used. Imaging is no substitute for a proper physical examination, which should be far more widely taught and practiced.

### What do we now need to know?

How will we judge if medical orthopaedics is a new discipline worthy of establishing in its own right? Some pertinent issues were helpfully set out in a paper of 1965 where the question of whether nuclear medicine is, or could be, a speciality in its own right, was articulated [9]. Other specialties have drifted into being chiefly because some interested practitioners have announced that the discipline is to exist and is, de facto, to be recognised, for example medical geology [10]. For medical orthopaedics, however, I believe that a more thoughtful, rigorous process will yield a better outcome, and an outcome more readily understood and adopted both in Israel and beyond.

What criteria might we need to make such a decision? Firstly, it is essential to assess the burden of illness which does not readily fit into any other discipline. Secondly, it is necessary to gather information on the evidence base for diagnosis and treatments and identify gaps where evidence is lacking. Thirdly, there must be benefit to a sizeable group of patients of adopting the model of a discipline focused on their conditions. Ideally the benefits would be both health and cost related. Fourthly, the case must be made for setting up a medical discipline rather than a discipline led by any other group of health professionals. As this commentary, and the paper by Finestone, are read and discussed, further criteria might be agreed.

Before deciding if a medical orthopaedics specialty is warranted, it will be essential to bring accurate facts and figures together. An appropriate group of clinicians should review the categories of presenting symptoms and signs which lead patients to seek help. The group should begin by agreeing, in as clearly defined detail as possible, a tried and tested rubric against which a cohort of cases can be systematically analysed. Ideally the group would not be limited to doctors with an interest in this area; rather, it would include physiotherapists, psychologists, occupational therapists, nurses and others whose work would allow them to contribute to the evidence gathering and analysis. I believe there is a case for including some patients, and certainly would support a debate on their best place within this work.

The group would be tasked with assembling solid evidence of the size of the challenge, and delineating it and its limits, so that any future specialty would be informed by the best and most current agreed intelligence. The rubric would best be developed and agreed internationally so that any medical orthopaedic specialty will grow harmoniously across the globe and readily compare data.

Other specialties have taken a great deal of time to reach this point, but there are good examples of the powerful importance of a careful process where it has happened. For example, the regular 5-year international review of evidence in emergency life support skills [11] published by the American Heart Association has undoubtedly allowed lives to be saved world-wide. The sharing of practice and experience has been invaluable in setting standards globally and on the continuous improvements of those standards.

Beyond being helpful to garner accurate, current data on the size and scale of the problem, it will be important to determine which conditions are generally successfully treated right now and which are not; and which treatments or advice provide relief from symptoms most cheaply, speedily and acceptably by patients, and which carry fewest adverse effects.

We know, for example, that there is a free market in orthotics, where the alchemy of the bronze plate of the surgeon’s consulting room can lend weight to the purchase of any suggested device, however scant the evidence for its effectiveness. The data gathering exercise discussed above will helpfully be complemented by a prospective study of the actual spend now on (say) orthotics. The value of the spend would help identify where funds might be drawn should there be agreement to establish a medical orthopaedic specialty. In a best case scenario, the resources for this specialty might be drawn from funds saved from ceasing non-evidence based investigations and interventions, be they imaging, any other tests, drugs, devices or other means intended to alleviate symptoms and improve function.

### What do we need to do once we have firm evidence?

Data alone will not address the issues. There is an important debate to be held on whether medical orthopaedics should be an independent medical specialty, or a physiotherapy domain, or one for primary care physicians with an interest in medical orthopaedics (as some general practitioners in the UK can be), or belongs with some other specialist group. It may be that a multi-disciplinary approach would be most successful. There is a need to include, for instance, the place of movement and exercise in remedying MSC. This might be a key element of treatments, and one where non-doctors such as physiotherapists have the greatest knowledge and skills. It would be very exciting to see a multi-disciplinary specialty where the leading experts were drawn from among doctors and others as equals. This would send strong signals about the sharing of key expertise between clinicians.

Debate should focus on fashioning a workable way forward which is evidence based, feasible, as inexpensive as possible, and using resources already within the systems we have as far as is possible. Again, an important debate is needed to articulate criteria against which progress to an improved approach to non-surgical MSC could be measured. The voices of patients should be present from the beginning and will help shape the priorities of any work in this arena. Patients have had real impact in treatments for many long term conditions such as type 2 diabetes, and pain management. Solutions should be acceptable to professional and patient alike.

Dr Finestone raises an important subject. I hope the ensuing debate starts quickly, is lively, and leads to evidenced actions to meet the challenge of better diagnosing and treating this large group of patients.

## Competing interests

The author declares that she has no competing interests.

## Author information

Dr Gillian Schiller is an independent health policy advisor from England. She has qualifications in health (MB BS), management (MBA) and health related law and ethics (LLM). Her work in the UK has included policy development at local, regional and national levels.

## Commentary on

Finestone AS, Vulfsons S, Milgrom C, Lahad A, Moshe S, Agar G, Greenberg D. The case for orthopaedic medicine in Israel. Isr J Health Policy Res 2013, 2:42.
